# Identification and phylogenetic analysis of *Enterococcus* isolates using MALDI-TOF MS and VITEK 2

**DOI:** 10.1186/s13568-023-01525-y

**Published:** 2023-02-22

**Authors:** Se-Hyung Kim, Jung-Whan Chon, Hyo-Won Jeong, Kwang-Young Song, Dong-Hyeon Kim, Dongryeoul Bae, Hyunsook Kim, Kun-Ho Seo

**Affiliations:** 1 Center for One Health, Department of Veterinary Public Health, College of Veterinary Medicine, Konkuk University, 120 Neungdong-ro, Gwangjin-gu, Seoul, 05029 Korea; 2 Seoul Metropolitan Government Research Institute of Public Health and Environment, Gyonggi-Do, 13818 Korea; 3 Department of Food and Nutrition, College of Human Ecology, Hanyang Univeristy, 222 Wangsimni-ro, Seongdong-gu, Seoul, 04763 Korea

**Keywords:** *Enterococcus*, Identification, MALDI-TOF MS, 16S rRNA, VITEK 2

## Abstract

The bacterial genus *Enterococcus* encompasses 38 species. Two of the most common species are *E. faecalis* and *E. faecium*. Recently, however, there has been an increase in clinical reports concerning less prevalent *Enterococcus* species, such as *E. durans, E. hirae,* and *E. gallinarum.* Rapid and accurate laboratory methods are needed to facilitate the identification of all these bacterial species. In the present study, we compared the relative accuracy of matrix-assisted laser desorption/ionisation time-of-flight mass spectrometry (MALDI-TOF MS), VITEK 2, and 16S rRNA gene sequencing using 39 enterococci isolates from dairy samples, and compared the resultant phylogenetic trees. We found that MALDI-TOF MS correctly identified all isolates at the species level except for one, whereas the VITEK 2 system, which is an automated identification system using biochemical characteristics of species, misidentified ten isolates. However, phylogenetic trees constructed from both methods showed all isolates in similar positions. Our results clearly showed that MALDI-TOF MS is a reliable and rapid tool for identifying *Enterococcus* species with greater discriminatory power than the biochemical assay method of VITEK 2.

## Key Points

- Although certain *Enterococcus* genus are used as starter cultures in many food products, this bacterial group is considered a human and animal pathogen which can cause nosocomical infections and urinary tract infections, and contribute to food spoilage.
- Accurate and reliable identification of *Enterococcus* genus present in food is of great importance for predicting shelf life and for managing and reducing microbiological food hazar.
- Phylogenetic trees obtained from MALDI-TOF MS and VITEK2 analyses were compared to evaluate the discriminative resolution of these two systems.

## Introduction

Genus *Enterococcus* is composed of 38 bacterial species, many of which can be isolated from various habitats, such as the feces of hospitalized patients, animals, animal-derived foods, and environments such as soil and water (Abriouel et al. 2008; Vu and Carvalho 2011; Quintela‐Baluja et al. 2013; Lebreton et al. 2014). Although certain enterococcal strains are used as starter cultures in many food products, this bacterial group is considered a human and animal pathogen which can cause nosocomical infections and urinary tract infections, and contribute to food spoilage (Vu and Carvalho 2011; Quintela‐Baluja et al. 2013; Lebreton et al. 2014). Along with their complicated virulence factors, enterococci have drawn renewed interest due to their ability to transfer antibiotic resistance genes to other pathogenic bacteria, which poses a great public health concern (Vu and Carvalho 2011; Lebreton et al. 2014). Further, the *Enterococcus* genus is not attributed the “generally recognized as safe” status, and is not included on the Qualified Presumption of Safety (QPS) list from the European Food Safety Authority (Hanchi et al. 2018).

Fast and reliable identification of bacterial species present in food is of great importance for predicting shelf life and for managing and reducing microbiological food hazards (Quintela‐Baluja et al. 2013). Many laboratories employ various diagnostic techniques to identify *Enterococcus* spp., including biochemical methods such as Analytical Profile Index (API) strips and the automated VITEK 2 system (bioMérieux, Marcy l’Etoile, France), and molecular approaches such as PCR-based 16S ribosomal RNA sequencing (Fang et al. 2012) and whole genome sequencing (Garza-Onzalez et al. 2020). Although phenotypic methods are regarded by food and clinical authorities in many countries as a gold standard by which to identify *Enterococcus* strains, these procedures are time-consuming and labor-intensive (Cheng et al. 1997). Further, species identification of enterococci using phenotypic methods is challenging due to the similar biochemical traits among species; therefore only a limited number of species can be identified (Quintela‐Baluja et al. 2013). Matrix-assisted laser desorption/ionisation time-of-flight mass spectrometry (MALDI-TOF MS) has gained popularity as an alternative identification method based on its rapid, cost-effective, and accurate ability to identify many pathogenic bacteria. However, this method has not been thoroughly tested in unusual *Enterococcus* species (Seng et al. 2009; Quintela‐Baluja et al. 2013). To the best of our knowledge, the applicability of MALDI-TOF MS to discriminating enterococci species from dairy sources has not been assessed, nor has a phylogenetic comparison of MALDI-TOF MS and VITEK 2 analyses been conducted. To conduct such an evaluation, we identified species of enterococci isolates from dairy samples using VITEK 2, MALDI-TOF MS, and 16S rRNA sequencing, and compared their discriminatory power at the species level. In addtion, phylogenetic trees obtained from MALDI-TOF MS and VITEK 2 analyses were compared to evaluate the discriminative resolution of these two systems.

## Materials and methods

### Bacterial strains

A total of 39 *Enterococcus* strains were recovered from dairy samples. The cells were stored at − 72 °C with 50% glycerol until ready for use. Bacteria were cultured on tryptic soy agar (TSA, Difco, Detroit, MI, USA) at 37 °C for 24 h prior to analysis. The strains *E. faecalis* KCTC 3206 and KCTC 3511, distributed by KCTC in South Korea, were used as controls.

### Biochemical phenotyping using VITEK 2 system

Biochemical reactions of the isolates were tested using the VITEK 2 system (bioMérieux) following the manufacturer’s instructions. In brief, pure colonies of each strain were cultured in TSA and suspended in sterile saline (0.45% NaCl) to a turbidity of McFarland 0.5–0.63. Bacteria were then injected into gram-positive (GP) cards, (bioMérieux) in order to conduct 42 biochemical tests using the following copmpounds: (AGAL = alpha-galactosidase; ADH1 = arginine dihydrolase; ADH2S = arginine dihydrolase; AGAL = alpha-galactosidase; AGLU = alpha-glucosidase; ALAA = alanine arylamidase; AMAN = alpha-mannosidase; AMY = amygdalin; APPA = ala-phe-pro arylamidase; ASPA = L-aspartic acid arylamidase; BACI = bacitracin resistance; BGAL = beta-galactosidase; BGUR = beta-glucorinidase; BGURR = beta glucorinidase; CDEX = alpha-cyclodextrin; DGAL = galactose; DMAL = maltose; DMAN = mannitol; DMNE = mannose; DRAF = raffinose; DRIB = ribose; DSOR = sorbitol; DTRE = trehalose; DXYL = xylose; LAC = lactose; LEUA = L-leucine arylamidase; LLATK = lactate; MBDG = methyl-beta-D-glucopyranoside; NAG = N-acetyl-glucosamine; NC6.5 = growth in 6.5 NaCl; NOVO = novobiocin resistance; O129R = O/129 resistance; OPTO = optochin resistance; PHOS = alkaline phosphatase; PIPLC = phosphatidylinositol-phospholipase C; POLYB = polymyxin B; PROA = pro arylamidase beta-glucuronidase; PUL = pullulan; PYRA = L-pyroglutamic acid arylamidase; SAC = sucrose; SAL = salicin; TYRA = tyrosine arylamidase; URE = urease). Results of this identification procedure were used to generate a phylogenetic tree using the unweighted pair group method with arithmetic mean (UPGMA) on the Molecular Evolutionary Genetics Analysis X (MEGA X) software (version 10.0.5).

### MALDI-TOF MS analysis

The isolates were prepared for MALDI-TOF MS analysis using the whole cell analysis as previously described (Fang et al. 2012; Deng et al. 2014), with modifications. In brief, each individual colony was incubated on TSA at 37 °C for 24 h, then transferred onto a MSP 96 target polished steel BC (Bruker Daltonik, Bremen, Germany) using a toothpick. To crystalize bacterial components, 1 μl of saturated α-cyano-4-hydroxy-cinnamic acid matrix solution in 50% acetonitrile-2.5% trifluoroacetic acid (Bruker Daltonik) was overlaid on each well and air-dried at room temperature (25 °C). Main spectrum profiles (MSP) of isolates were obtained using the microflex LT mass spectrometer (Bruker Daltonik) with default MSP identification standard settings (linear positive mode, 2000 to 20,000 Da). Bacteria were identified, and a phylogenetic tree was generated by analyzing MS spectra using MALDI Biotyper software (version 3.1) and flexControl software (version 3.4.127.0).

### 16S rRNA gene sequencing

The bacterial genomic DNA was extracted using NucliSENS EasyMag (bioMérieux) according to the manufacturer’s instructions. Sequences of the 16S rRNA gene of the isolates were generated by Bionics (Seoul, South Korea) using universial primers (27F, 5’-AGAGTTTGATCMTGGCTCAG-3’, and 1492R, 5’-GGTTACCTTGTTACGACTT-3’). Each of the raw 16S rRNA gene sequencing datasets was aligned using MEGA X software (version 10.0.5); species were identified using the NCBI BLASTn program (http://blast.ncbi.nlm.nih.gov). The results from 16S rRNA gene sequences with the homologous rate of above 99% were used as the standard for interpreting the results from VITEK 2 and MALDI-TOF MS analyses.

### Statistical analysis

Statistical analysis was performed using GraphPad InStat 3 software (San Diego, CA, USA) and data were analyzed using the two-sided Fisher’s exact test.

## Results

**Fig. 1:**
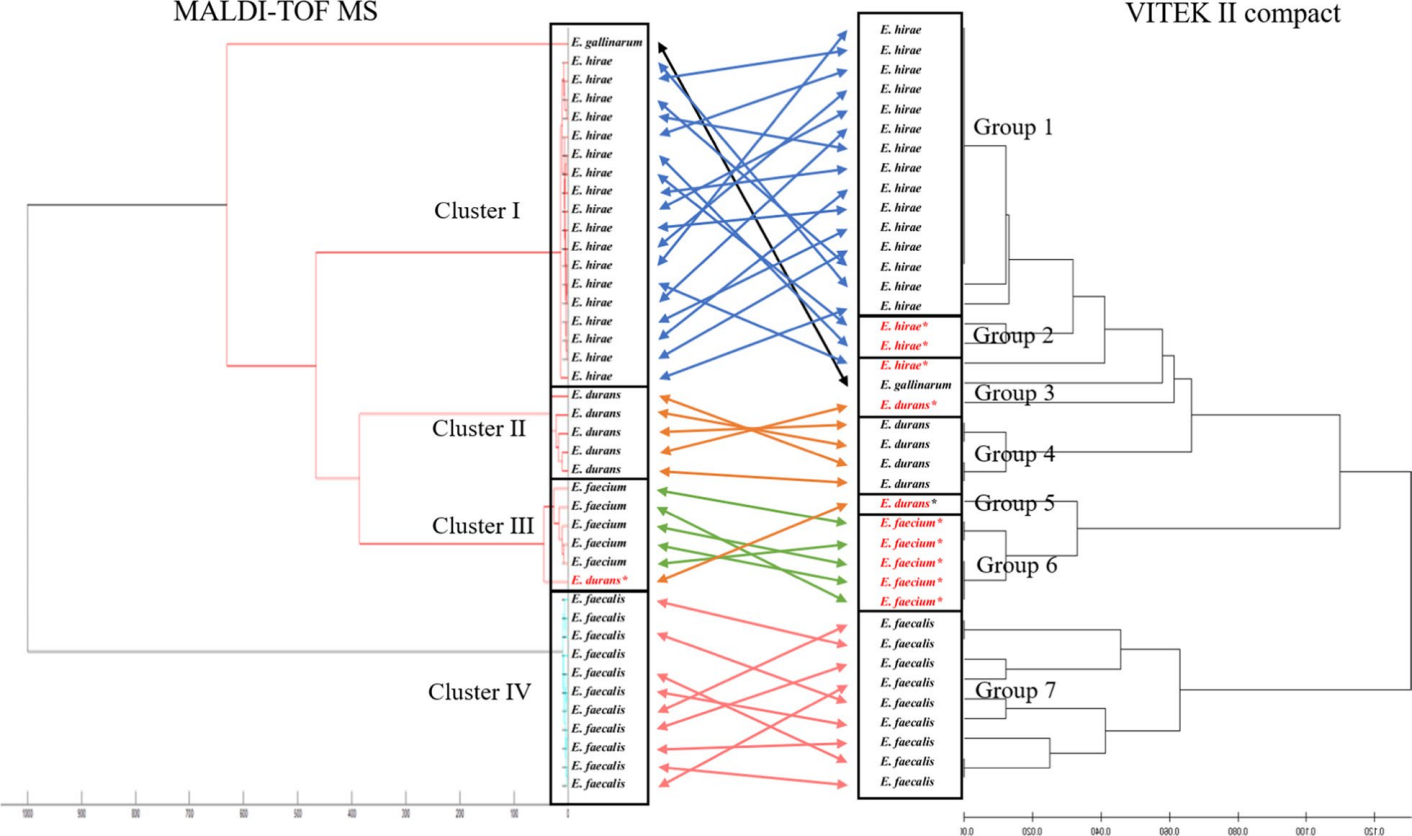
Comparison of phylogenetic trees obtained from MALDI-TOF MS spectra (left) and VITEK 2 biochemical patterns (right). Species were interpreted using the results from 16S rRNA sequencing. The arrows indicate the same colony or isolate used for both identification instrument. The species names illustrated are from 16S rRNA sequencing. Therefore, the species names species names with asterisk (*) from 16S rRNA sequencing indicates misidentified isolates by MALDI-TOF MS or VITEK 2

In this study, 16S rRNA sequencing revealed that the 39 enterococci isolates comprised 9 isolates of *E. faecalis* (23%), 5 of *E. faecium* (13%), 6 of *E. durans* (15%), 18 of *E. hirae* (46%), and 1 of *E. gallinarum* (3%). The results would be considered valid if the similarity values with type strains was above 99%. We compared sequencing results with those from MALDI-TOF MS and VITEK 2. In brief, a single *E. durans* isolate was misidentified as *E. faecium* by MALDI-TOF MS, whereas ten isolates were mismatched by VITEK 2 (Table 1) when log scale ≥ 2.0 for MALDI-TOF MS and identification probability ≥ 94% for VITEK 2 with reference strains were used. The species misidentified by VITEK 2 were as follows: two out of six *E. durans* isolates were misidentified as *E. gallinarum* and *E. faecium*, and three out of the eighteen *E. hirae* isolates were misidentified as *E. durans* and one as *E. gallinarum*. All of *E. faecium* isolates were misidentified as *E. durans*. These results demonstrate that the discriminative capability of MALDI-TOF MS is superior to that of VITEK 2 (*p* < 0.05). However, *E. faecalis* and *E. gallinarum* were correctly identified in both methods. Phylogenetic trees generated from MALDI-TOF MS and VITEK 2 analyses are shown in Fig. 1. The isolates were grouped into four clusters by MALDI-TOF MS, which revealed that each cluster was composed of specific species except for a single *E. gallinarum* isolate in Cluster I. In contrast, the isolates were clustered into the total of seven groups by VITEK 2 based on the biochemical patterns by which it designates species. The phylogenetic tree based on the latter system showed isolates in the same species adjacent to each other; however, they were classified as different species. For example, 11 isolates which were identified as *E. durans* were not clustered in the same group.

**Table 1:** Comparison of correct identification rates of *Enterococcus* isolates from MALDI-TOF MS and VITEK 2 at species level

16S rRNA species	Number (%) of correct identifications^a^	
“Gold standard”	MALDI-TOF MS	VITEK 2
*Enterococcus faecalis* (n = 9)	9 (100)	9 (100)
*Enterococcus faecium* (n = 5)	5 (100)	0 (0)
*Enterococcus durans* (n = 6)	5 (83)	4 (67)
*Enterococcus hirae* (n = 18)	18 (100)	15 (83)
*Enterococcus gallinarum* (n = 1)	1 (100)	1 (100)
Total (n = 39)	38 (97) A	29 (74) B

A log scale ≥ 2.0 for MALDI-TOF MS, the identificate rate above 94% for VITEK 2, and the similarity values with type strains above 99% were used as a determinant of correct identificatio to the species level

^a^Different letters (A, B) within a row indicate significant differences (*p* < 0.01)

## Discussion

Enterococci are ubiquitous in the environment; they can grow in the intestinal tracts of humans and animals (Zaheer et al. 2020). They cause numerous infections, including urinary tract infections and endocarditis, as well as various nosocomial infections (Fiore et al. 2019). In order to curb the spread of such diseases, it is important to identify enterococci at the species level in diagnostic laboratories (Vu and Carvalho 2011; Kirschner et al. 2001). Further, with the recent increase of clinical cases in which less common species, such as *E. durans*, *E. hirae*, *E. gallinarum* and *E. casseliflavus*, are implicated (Hammerum 2012; Zaheer et al. 2020), rapid and accurate identification at the species or subspecies level is necessary for the control of infections and the study of epidemiology (Willey et al. 1999; Kirschner et al. 2001).

Similar results were observed in previous studies, in which MALDI-TOF MS correctly identified all clinical isolates tested, including *E. faecalis*, *E. faecium*, *E. casseliflavus*, and *E. gallinarum*. In contrast, VITEK 2 misidentified 10 of 131 isolates (Fang et al. 2012). Such misidentification may reflect unreliable identification of uncommon *Enterococcus* species, or the occurance of atypical phenotypic properties (Singer et al. 1996; Tsakris et al. 1998). This suggests that the VITEK 2 database needs further improvement to increase accuracy of *Enterococcus* species identification. Quintela‐Baluja et al. (2013) revealed that enterococci can have different mass spectral profiles, depending on whether they originate from dairy or meat (Quintela‐Baluja et al. 2013). Therefore, to ensure accurate species identification, enterococci originating from animal-derived sources should be evaluated by MALDI-TOF MS. This study is the first to assess the discriminative power of MALDI-TOF MS in relation to common and uncommon *Enterococcus* species from dairy samples, including *E. durans*, *E. hirae*, and *E. gallinarum*. Although the source of the isolates in our study was dairy samples, our results indicate that MALDI-TOF MS could be a useful alternative diagnostic tool for screening enterococci species not only from foods, but also in etiological clinical applications which require fast and reliable species- or strain-level identification.

Although each method differentiates based on different characteristics (i.e., protein spectrum versus biochemical patterns) (Sala-Comorera et al. 2016), the phylogenetic trees obtained from the two systems placed the isolates in identical positions (Fig. 1). In accordance with our results, Sala-Comorer et al. (2016) demonstrated that dendrograms obtained from MALDI-TOF MS showed taxonomic resolution similar to that of the PhenePlate™ system, a biochemical phenotyping method (Sala-Comorer et al. 2016). These results suggest that although both methods display similar levels of resolution in discriminating the isolates, MALDI-TOF MS is a much more accurate method of species identification than VITEK 2.

In the present study, we compared two bacterial diagnostic techniques which are currently used in laboratories, VITEK 2 and MALDI-TOF MS. The latter has been used as an alternative tool for identification and phylogenic study of enterococci (Stępień-Pyśniak et al. 2017). Our evaluation of the accuracy of each system in identifying species among *Enterococcus* isolates showed MALDI-TOF MS to be more accurate than VITEK 2. Whereas MALDI-TOF MS misidentified only a single *E. durans* isolate, VITEK 2 showed less discriminative capability regarding both common (*E. faecium*) and uncommon species (*E. durans* and *E. hirae*). An excess or a lack of quantity of the sample when the sample was deposited on the target plate could interfere with the result. These results imply that MALDI-TOF MS can be used as fast and reliable tool for bacterial identification, and for analyzing phylogenetic relationships within genera.

In addition to its high level of accuracy, MALDI-TOF MS was the most time- and cost-effective method among various other automated biochemical identification methods, such as API and the VITEK system (Seng et al. 2009). A previous study similarly showed that MALDI-TOF MS is more efficient than VITEK 2 in identifying *Enterococcus* species other than *faecalis* and *faecium* (Fang et al. 2012). Because the results of MALDI-TOF MS are based on a software-installed database of mass spectral profiles, this method requires more extensive spectrum data than other systems. However, its reliability is superior; 38 out of 39 *Enterococcus* isolates in this study were accurately identified.

In conclusion, the results of this study indicate that MALDI-TOF MS can be used for routine identification of both usual and unusual enterococci from dairy products, as it shows greater species-level discriminatory power than VITEK 2. Further, MALDI-TOF MS is cost-effective and requires less time to identify the bacteria, making it a useful technique for diagnostic laboratories which need rapid identification of bacterial species. However, one limitation to this study is that we only tested for species in the *Enterococcus* genus, and have not tested the sensitivity of MALDI-TOF MS to species of other genera. Further study is needed with a larger number of isolates for each species because the number of strains for each species was too small as to be able to make a clear statement about the value of the individual identification method.

## Data Availability

The raw sequencing data in this study were deposited in the NCBI Sequence Read Archive (SRA) under accession number SUB8439660.
